# Extreme Events and Event Size Fluctuations in Resetting Random Walks on Networks

**DOI:** 10.3390/e27121215

**Published:** 2025-11-28

**Authors:** Xiaohan Sun, Shaoxiang Zhu, Anlin Li

**Affiliations:** 1 School of Mathematical Science, Jiangsu University, Zhenjiang 212013, China; njsunxiaohan@126.com (X.S.);; 2 School of Mechanical Engineering, Jiangsu University, Zhenjiang 212013, China

**Keywords:** extreme events, random walks, stochastic resetting

## Abstract

Random walks with stochastic resetting, where walkers periodically return to a designated node, have emerged as an important framework for understanding transport processes in complex networks. While resetting is known to optimize search times, its effects on extreme events—defined as exceedances of walker flux above a critical threshold—remain largely unexplored. Such events model critical network phenomena, including traffic congestion, server overloads, and infrastructure failures. In this work, we systematically investigate how stochastic resetting influences both the probability and magnitude of extreme events in complex networks. Through analytical derivation of the stationary occupation probabilities and comprehensive numerical simulations, we demonstrate that resetting significantly reduces the occurrence of extreme events while concentrating event-size fluctuations. Our results reveal a universal suppression effect: increasing the resetting rate γ monotonically decreases extreme event probabilities across all nodes, with complete elimination at γ=1. Notably, this suppression is most pronounced for vulnerable low-degree nodes and nodes distant from the resetting node, which experience the largest reduction in both event probability and fluctuation magnitude. These findings provide theoretical foundations for using resetting as a control mechanism to mitigate extreme events in networked systems.

## 1. Introduction

Extreme events (EEs) are typically observed in many geophyscial phenomena such as the earthquakes, cyclones, droughts and so on [1,2,3]. These represent extreme events recorded in univariate time series, and a large amount of theoretical and practical results have been obtained regarding the statistical and dynamic analysis of these extreme events. [4,5] However, as early as a century ago, extreme events in univariate time seriesbarose in the research of classical extreme value theory, the extreme events in complex networks have only attracted attention in recent years [6,7,8]. Extreme events taking place on complex networks is a fairly common occurrence, such as traffic jam in roads and other transportation networks, web servers not responding due to the heavy load of http requests, floods in the network of rivers, power black outs due to tripping of power grids, etc. [9,10]. Though such events typically have low probability of occurrence, they are regarded as significant owing to the social and financial losses suffered on account of most of these extreme events. Generally, these physical phenomena can be regarded as an emergent phenomena arising from flux on networks and could be regarded as extreme events arising primarily because of the limited handling capacity of the node [11,12]. However, an extreme event, defined as exceedances above the threshold, is not only affected by the capacity of the node, but is also attributable to natural fluctuations passing through a node. When the fluctuation in the flux passing through a node exceeds a prescribed threshold, extreme events will occur accordingly [6,13,14].

The analysis model we introduce in this research is the random walk on complex networks [15]. Random walks are ubiquitous in nature and have applications in a broad range of fields [16,17,18]. Recent research has shown that complex network exploration using random walks can be defined in terms of moving to nearest-neighbor nodes or as long-range hops between distant nodes [19,20]. Random walks are also interesting as a mechanism of transport and search within networks [21,22]. Recently, stochastic resetting has emerged as a significant concept in understanding a variety of random walk processes on complex networks [23,24,25,26,27,28]. This mechanism, which involves reinitializing a random walk to a particular node, has been explored to control and optimize diffusion-based search dynamical processes [29,30]. Actually, Stochastic resetting, as a classic research topic in the field of non-equilibrium statistical physics, traces its theoretical roots to earlier investigations of stochastic processes. As early as the 1970s through the early 2000s, this concept had emerged in academic literature under diverse denominations, including killing processes, birth-death processes, the theory of catastrophes, and the theory of economic crashes [31,32,33,34,35]. However, due to the “short-term memory effect” within the academic community, these foundational early works have gradually fallen into neglect. In recent years, Pal and Madjumdar et al. have made significant contributions to the understanding of resetting dynamics, first-passage under resetting, and diffusion with stochastic resetting [36,37,38,39], bringing the stochastic resetting theory back into the spotlight of the academic community. Furthermore, Sokolov et al. have conducted a series of studies on stochastic resetting in fractional Brownian motion and geometric Brownian motion [40,41,42,43], systematically delineating the theory’s historical context and tracing it back to the aforementioned original stochastic process research—thereby furnishing a crucial reference for clarifying the academic lineage. Meanwhile, their pioneering contributions have solidified their status as the true originators in the field of non-equilibrium statistical physics. The effect of stochastic resetting on network-based processes, especially random walks, has implications for a range of applications, from search algorithms to biological transport phenomena [44,45]. Recent contributions relating to optimal reset protocols further emphasize the importance of tuning the reset conditions to achieve specific goals, such as minimizing the search time or optimizing resource allocation on the network [46,47]. By optimizing the reset protocol, the efficiency of random walks on complex networks can be expedited.

In this work, we model the transport process as resetting random walks on networks and delve into the study of the probability for EEs and event size fluctuations. Relating to how the connectivity of the network affects the probability for the extreme event occurrence, by modeling the transport as the standard random walk, the V. Kishore has displayed in Ref. [6] that the probability for the occurrence of EEs, which depends only on the stationary distribution of a node arising due to inherent fluctuations. In our research, the threshold was chosen to be proportional to typical fluctuation size on *i*-th node and the extreme events are identified after taking care of the natural variability in the flux passing through the given node. Furthermore, it was shown that the probability for EEs is higher for small degree nodes than for hubs or large degree nodes. This hints that the large flux does not translate into extreme events and even though hubs or large degree nodes draw large flux they are less liable to extreme events on a connected network. By leveraging the stochastic resetting, there is a surprising result that the stochastic resetting can reduce the probability of EEs occuring and event size fluctuations in our research. And our work can be generalized to study the impact of stochastic resetting on complex networks. Specifically, we investigate the impact of stochastic resetting on the probability for EEs and event size fluctuations within the Barabási-Albert network [48], which exhibits scale-free characteristics.

The remainder of this paper is organized as follows. Section 2 introduces random walks on networks with stochastic resetting. Section 3 describes the probability for extreme events with stochastic resetting. Section 4 elaborates fluctuations in event size with stochastic resetting. Section 5 summarizes the conclusions derived from this research.

## 2. Random Walk on Networks with Stochastic Resetting

The theoretical framework for random walks on networks with stochastic resetting presented in this section follows established formalisms. In particular, the derivation of the stationary occupation probability was first introduced in Ref. [23] and has since been re-derived using alternative approaches, such as the renewal method [49,50]. Our presentation below is consistent with these foundational works that they all utilize the eigenvalue and eigenvector of the transition matrix without resetting. The difference lies in the fact that the master equation exhibited by time-dependent resetting mechanism and resetting mechanism proposed in this paper are different, resulting in certain discrepancies between the final results.

A connected, undirected network with *N* nodes and *E* edges is considered. Its connectivity structure is determined by an adjacency matrix *A* whose element $A_{ij}=1$ if nodes *i* and *j* are connected by an edge, and is 0 otherwise.

On this network structure, diffusion of *W* independent walkers is considered who perform random walks in the sense explained below. For convenience, we assume that a random walker is initially located at node *i* at t=0 in order to define the transition probability. However, it should be emphasized that when discussing the stationary distribution the specific choice of the initial position is irrelevant, since the resetting dynamics continuously drives the walker to the designated reset node *r*.

The random walker performs at t=1, 2, 3, … in two types of steps: a hop to one of the neighbors of the node presently occupied with probability 1−γ or a resetting to a given node *r* with probability γ.

Without resetting (γ=0), the probability that the random walker hops from *i* to *j* is $b_{i\to j}=\frac{A_{ij}}{k_{i}}\;\left(i,\;j=1,\;2,\;\ldots ,\;N\right)$ which is the element of the transition matrix *B*, depicting the random walk without resetting and possessing the characteristic of normalization (each row of *B* adds up to 1). Thus, the time evolution of probability is given by the master equation [23,24]:

(1) $$P_{ij}\left(t+1;r,\gamma \right)=\left(1-\gamma \right)\sum_{l=1}^{N}P_{il}\left(t;r,\gamma \right)b_{l\to j}+\gamma \delta_{rj},$$

where $P_{ij}\left(t+1;r,\gamma \right)$ denotes the probability of the random walker starting from node *i* and reaching node *j* at time *t* given the resetting node *r* and the resetting rate γ. The first term of the equation represents the walker hoping to the nearest nodes from current position, and the second term indicates resetting to the given node (where $\delta_{rj}$ is the Kronecker delta). Let us define Π(r,γ) as the transition matrix with elements $\pi_{l\to j}\left(r,\gamma \right)=\left(1-\gamma \right)b_{l\to j}+\gamma \delta_{rj}$. Thus, the master equation can be rewritten as follows:

(2) $$P_{ij}\left(t+1;r,\gamma \right)=\sum_{l=1}^{N}P_{il}\left(t;r,\gamma \right)\pi_{l\to j}\left(r,\gamma \right).$$

The form of Equation (2) satisfies the property of a Markov chain, which means $\sum_{j=1}^{N}\pi_{i\to j}\left(r,\gamma \right)=1.$

Like *B*, the matrix Π(r,γ) is also a stochastic matrix: knowing its eigenvalues and eigenvectors expedites us to calculate the occupation probability at any time, even including the stationary distribution.

Owing to the fact that the matrix Π(r,γ) is related to *B*, we can obtain the relationship between the two stochastic matrices. We denote the eigenvalues of the matrix *B* as $\lambda_{i}$, and its left and right eigenvectors are denoted as $\langle {\bar{\varphi }}_{i}|$ and $|\varphi_{i}\rangle$ respectively, for i=1, 2, …, N.

Similarly, the eigenvalues of Π(r,γ) are denoted as $\zeta_{i}\left(r,\gamma \right)$ and its eigenvectors are denoted as $\langle {\bar{\psi }}_{i}\left(r,\gamma \right)|$ and $|\psi_{i}\left(r,\gamma \right)\rangle$. Based on the master equation, we import the following identity:

(3) Π(r,γ)=(1−γ)B+γΨ(r),

where the elements of the matrix Ψ(r) are $\Psi_{lm}\left(r\right)=\delta_{mr}$; so, the matrix has entries 1 in the $r^{\mathrm{th}}$-column and null entries everywhere else. Then the $\zeta_{i}\left(r,\gamma \right)$ can be described as follows [23]:

(4) $$\zeta_{i}\left(r,\gamma \right)=\left\{\begin{array}{cc} 1 & \mathrm{for}\;i=1\\ \left(1-\gamma \right)\lambda_{i} & \mathrm{for}\;i=2,\;3,\;\ldots ,\;N. \end{array}\right.$$

Furthermore, the left and right eigenvectors of Π(r,γ) are described as follows:

(5) $$\langle {\bar{\psi }}_{1}\left(r,\gamma \right)|=\langle {\bar{\varphi }}_{1}|+\sum_{i=2}^{N}\frac{\gamma }{1-\left(1-\gamma \right)\lambda_{i}}\frac{\langle r|\varphi_{i}\rangle }{\langle r|\varphi_{i}\rangle }\langle {\bar{\varphi }}_{i}|,$$

where $\langle {\bar{\psi }}_{i}\left(r,\gamma \right)|=\langle {\bar{\varphi }}_{i}|$ for i=2, 3, …, N. Similarly, the right eigenvectors are given by

(6) $$|\psi_{i}\left(r,\gamma \right)\rangle =|\varphi_{i}\rangle +\frac{\gamma }{1-\left(1-\gamma \right)\lambda_{i}}\frac{\langle r|\varphi_{i}\rangle }{\langle r|\varphi_{1}\rangle }|\varphi_{1}\rangle ,$$

for i=2, 3, …, N, and $|\psi_{1}\left(r,\gamma \right)\rangle =|\varphi_{1}\rangle$. |r〉 is defined as the vector whose elements equal to 0 except the *r*-th one, which equals to 1. With the left and right eigenvectors at hand, the matrix Π(r,γ) can be characterized by the following spectral representation.

(7) $$\Pi \left(r,\gamma \right)=\sum_{i=1}^{N}\zeta_{i}\left(r,\gamma \right)|\psi_{i}\left(r,\gamma \right)\rangle \langle {\bar{\psi }}_{i}\left(r,\gamma \right)|.$$

Within the discrete step, the master equation of the occupation probability can be rewritten as

(8) $$P_{ij}\left(t;r,\gamma \right)={\langle i|\Pi \left(r,\gamma \right)}^{t}|j\rangle ,$$

Using the spectral representation, this can be restated as follows:

(9) $$P_{ij}\left(t;r,\gamma \right)=\langle i|\psi_{1}\left(r,\gamma \right)\rangle \langle {\bar{\psi }}_{1}\left(r,\gamma \right)|j\rangle +\sum_{l=2}^{N}{\left[\left(1-\gamma \right)\lambda_{l}\right]}^{t}\langle i|\psi_{l}\left(r,\gamma \right)\rangle \langle {\bar{\psi }}_{l}\left(r,\gamma \right)|j\rangle ,$$

where the stationary distribution, which we define as $p_{j}\left(r,\gamma \right)$, is equal to the first term of Equation (9). Utilizing the expression of the left and right eigenvectors of Π(r,γ), the stationary distribution is given by

(10) $$p_{j}\left(r,\gamma \right)=\frac{k_{j}}{\sum_{m=1}^{N}k_{m}}+\gamma \sum_{l=2}^{N}\frac{\langle r|\varphi_{l}\rangle \langle {\bar{\varphi }}_{l}|j\rangle }{1-\left(1-\gamma \right)\lambda_{l}},$$

where $\langle i|\varphi_{1}\rangle \langle {\bar{\varphi }}_{1}|j\rangle =\frac{k_{j}}{\sum_{m=1}^{N}k_{m}}$ is the stationary distribution of the random walk without resetting. The second term of Equation (10) is the consequence of the resetting dynamical process, which disrupts the equilibrium of the general distribution.

## 3. Probability for Extreme Events with Stochastic Resetting

In this section, we focus on the probability for EEs with stochastic resetting. First of all, we consider the distribution of the number of random walkers at a given node. We assume the dynamical process that there are a total of *W* random walkers wandering in the network with stochastic resetting. We define the probability of finding *w* random walkers at node *i* (the flux passing through a node) where the resetting node is *r* and the resetting rate is γ as $f_{i}\left(w,r,\gamma \right)$. It is obvious that the walkers are relatively independent when performing the random walk process which leads to the variable *w* follows the binomial distribution. Thus, the probability $f_{i}\left(w,r,\gamma \right)$ is obtained by

(11) fi(w;r,γ)=Wwpi(r,γ)w[1−pi(r,γ)]W−w.

We can also derive the mean and standard deviation of finding *w* walkers at a given node as follows:

(12) $$\begin{array}{c} \langle f_{i}\left(r,\gamma \right)\rangle =Wp_{i}\left(r,\gamma \right),\\ \;\\ \;\;\;\sigma_{i}\left(r,\gamma \right)=\sqrt{Wp_{i}\left(r,\gamma \right)\left[1-p_{i}\left(r,\gamma \right)\right]}. \end{array}$$

We can observe that the relationship between the mean and standard deviation can be approximately expressed as $\sigma_{i}{\left(r,\gamma \right)}^{2}\approx \langle f_{i}\left(r,\gamma \right)\rangle$ for non-resetting nodes owing to the value of $p_{i}\left(r,\gamma \right)$ being much smaller than 1. However, this approximation breaks down for resetting nodes, particularly at large resetting rates γ, where the occupation probability becomes significant. This relation can be thought of as a generalization of a similar relation for the standard random walks reported in Ref. [14]. Moreover, the expressions in Equations (11) and (12) depend only on the stationary distribution $p_{i}\left(r,\gamma \right)$ characterizing each node, and are independent of the network topology, making them applicable to different types of networks, such as scale-free networks, random networks, or fractal networks.

**Figure 1:**
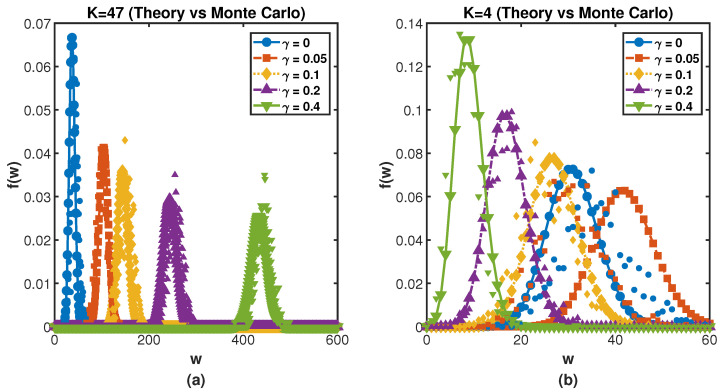
Comparison of theoretical predictions and Monte Carlo simulations for the distribution of random walkers visiting (**a**) the resetting node with degree k=47 and (**b**) a non-resetting node with degree k=4. The solid lines represent the theoretical binomial distributions derived from the stationary occupation probabilities (Equation (11)), while the markers show the results from Monte Carlo simulations with N=1000 steps over 1000 independent realizations. Different colors correspond to different resetting probabilities γ=0, 0.05, 0.1, 0.2, 0.4. The excellent agreement between theory and simulations validates our analytical framework.

Figure 1 presents a comprehensive comparison between theoretical predictions and Monte Carlo simulations for the distribution of random walkers in a scale-free network. The solid lines represent the theoretical binomial distributions derived from Equation (11), while the markers show the results from extensive Monte Carlo simulations with 1000 independent realizations. In panel (a), we show the distribution at a resetting node with degree k=47 (the network hub), and panel (b) displays the distribution at a non-resetting node with degree k=4. Different colors correspond to resetting rates γ=0, 0.05, 0.1, 0.2, 0.4. The remarkable agreement between theory and simulations across all resetting rates validates our analytical framework and demonstrates the accuracy of the stationary distribution predictions.

As clearly demonstrated in Figure 1, the resetting strategy exhibits fundamentally different effects on hub nodes and peripheral nodes. In panel (a), increasing the resetting rate γ significantly sharpens the distribution and increases the probability of finding large numbers of random walkers at the central hub node. Conversely, panel (b) shows that resetting broadens the distribution and reduces the occurrence probability of large walker numbers at low-degree peripheral nodes. This contrasting behavior underscores the crucial role of network centrality in determining the response to stochastic resetting. These research results provide important insights for understanding the probability distribution of extreme events with stochastic resetting and the fluctuations in event size in subsequent research.

We determine that the the probabilities for the occurrence of EEs are very small, which are reflected at the tails of the binomial distribution. We apply this principle to each node in the BA scale-free network with the resetting node *r* and the resetting rate γ. As mentioned above, at any given time, the number of random walkers *w* passing through a node in the network follows the binomial distribution given by Equation (11), an extreme event is considered to occur at a node if more than *q* random walkers pass through it, i.e., w≥q. Therefore, the probability of an extreme event can be expressed as follows:

(13) Fi(r,γ)=∑w=qiWWwpi(r,γ)w(1−pi(r,γ))W−w.

It should be noted that the scenario where the threshold $q_{i}$ is constant adds no value to our research. Thus, we suggest that a more meaningful and realistic approach is to define the threshold based on the natural variability in the flux passing through a node. Therefore, we define the threshold for extreme events as follows [14]:

(14) $$q_{i}=\langle f_{i}\left(r,\gamma \right)\rangle +m\sigma_{i}\left(r,\gamma \right).$$

**Figure 2:**
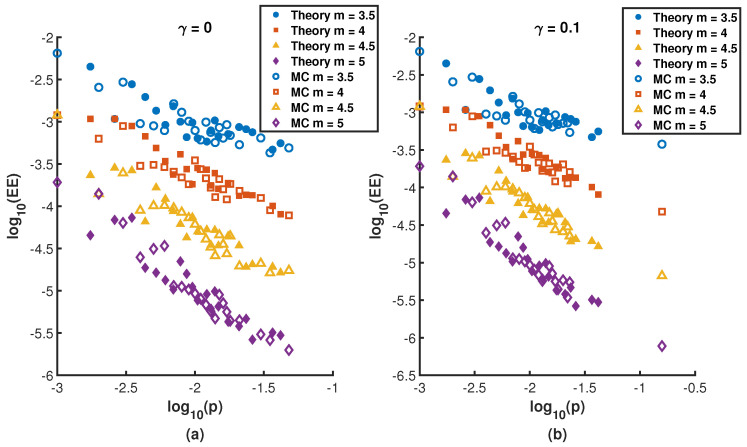
The probability of EEs ($F_{i}\left(r,\gamma \right)$) as a function of stationary distribution $p_{i}\left(r,\gamma \right)$ for different values of *m* when γ=0 and γ=0.1 in log-log plot, superimposing the theoretical values and simulated values for comparison. (**a**) γ=0; (**b**) γ=0.1.

**Figure 3:**
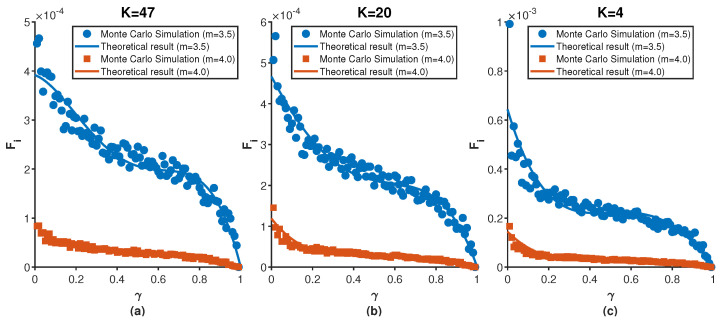
The probability of EEs ($F_{i}\left(r,\gamma \right)$) for nodes of different degrees as the resetting nodes, with *m* taking values of 3.0, 3.5, and 4.0. The circular markers represent simulation results on the BA scale-free network, while the curves represent the theoretical analysis obtained from Equation (13). (**a**) Results when the resetting node has degree K=47; (**b**) Results when the resetting node has degree K=20; (**c**) Results when the resetting node has degree K=4.

We consider m≥0, and the standard deviation $\sigma_{i}\left(r,\gamma \right)$ and the mean $\langle f_{i}\left(r,\gamma \right)\rangle$ are given by Equation (12). Based on Equation (13), we can intuitively analyze that the probability of EE ($F_{i}\left(r,\gamma \right)$) is directly determined by the stationary distribution $p_{i}\left(r,\gamma \right)$. Figure 2 vividly presents the impact of different values of $p_{i}\left(r,\gamma \right)$ on the probability of EEs occurring. Both Figure 2a,b show that the the nodes with large values of $p_{i}\left(r,\gamma \right)$ are less likely to experience extreme events and resetting can reduce $F_{i}\left(r,\gamma \right)$ occurring to a certain extent by comparing Figure 2a,b. Meanwhile, the extreme events with large values of parameter *m* are less likely to occur analyzed from the Figure 2. By substituting Equation (14) into Equation (13), we find that the probability of EEs mainly depends on the resetting rate γ, the parameter *m* and the total number of random walkers *W*. However, once given $p_{i}\left(r,\gamma \right)$, the extreme events are a direct consequence of the shape of the binomial distribution independently of the walker evolution. Furthermore, in particular in the limit of large *W*, the central limit theorem holds and the binomial distribution of *w* converge to the Gaussian distribution that fully determines the value of the probability of EEs, independently of $p_{i}\left(r,\gamma \right)$ which can be confirmed from Figure 1. Actually, based on the large deviation principle [51], the probability $f_{i}\left(w;r,\gamma \right)$ can be characterized by the continuous rate function J(w) that $f_{i}\left(w;r,\gamma \right)\approx e^{-nJ(w)}$ and $F_{i}\left(r,\gamma \right)$ can be restated as integral form $\int_{q_{i}}^{\infty }e^{-nJ(w)}dw$ (*n* is a integer parameter assumed to be large), which makes significant contributions to determine the $f_{i}\left(w;r,\gamma \right)$ whether *W* is relatively large or small and is conducive to further analyze the probability of EEs under different values of stationary distribution $p_{i}\left(r,\gamma \right)$. Figure 3 presents $F_{i}\left(r,\gamma \right)$ for different resetting nodes *i* as the resetting rate γ. It is worth noting that $p_{i}\left(r,\gamma \right)$ is the increasing function of γ when *i* is defined as the resetting nodes. Figure 3a–c are based on simulations conducted on the BA scale-free network consisting of 200 nodes, 780 edges, and 1560 random walkers. The simulation results show excellent agreement with the analytical distribution obtained from Equation (13). The simulation results shown in Figure 3 are obtained by averaging over 50 independent runs.

By examining Figure 3c, we can intuitively obtain that the probability of EEs on the low-degree resetting node in the BA network decreases significantly as the resetting rate γ increases. At the same time, as the parameter *m* increases, the threshold $q_{i}$ also increases, leading to a lower probability of EEs for the node (k=4). This is consistent with the analytical results derived from Equation (13). The same conclusion applies to Figure 3a,b as well. Moreover, even if a large amount of random walkers passing through the central node (k=47), the probability for the occurrence of extreme events is lower than that in nodes with a lower degree than it by comparing Figure 3a–c. Without loss of generality, we draw a conclusion that the probabilities of EEs across all nodes in the BA scale-free network decrease as the resetting rate γ increases.

**Figure 4:**
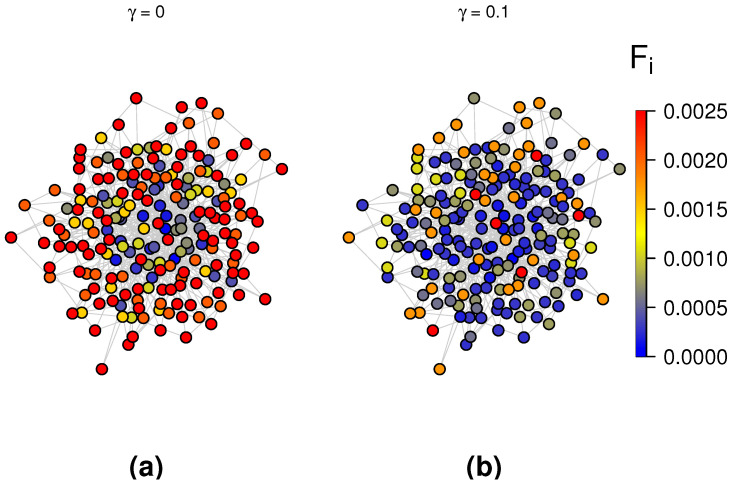
The probability distribution of EEs in the BA scale-free network without resetting compared to the BA scale-free network with resetting (where the central node is the resetting node and the resetting rate γ=0.1). (**a**) γ=0; (**b**) γ=0.1.

**Figure 5:**
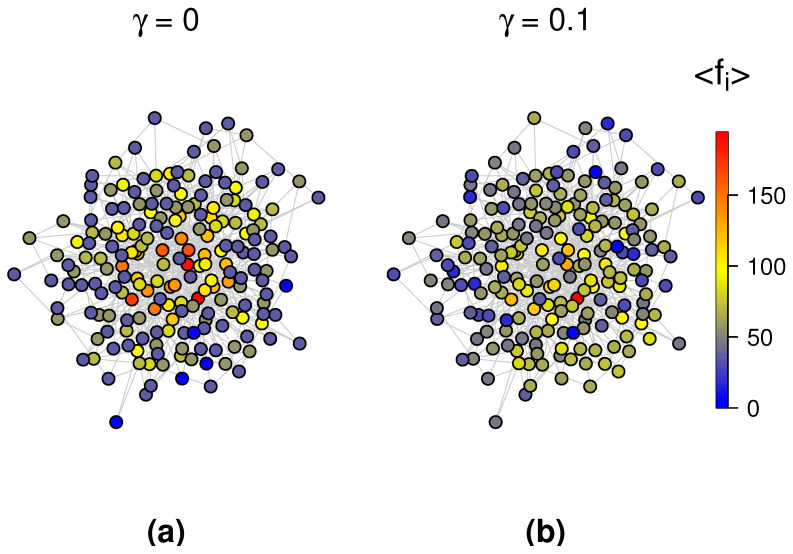
The distribution of average occupation $\langle f_{i}\left(r,\gamma \right)\rangle$ in the BA scale-free network with resetting rates γ=0 and γ=0.1. (**a**) γ=0; (**b**) γ=0.1.

To further validate the conclusions drawn from Figure 3 and to investigate the effect of stochastic resetting on extreme events, we compared the probability of EEs under standard random walks and random walks with resetting. Figure 4 presents the probability of EEs across all nodes in the BA scale-free network consisting of 100 nodes, 562 edges, and the results are performed by 1124 random walkers. The left panel corresponds to the case without resetting node, while the right panel shows the case where the central node serves as the resetting node with a resetting rate of γ=0.1. The color change of the energy bar represents the change in the probability for the occurrence of EEs: warmer colors indicate a higher probability of extreme events occurring at a node, while cooler colors indicate a lower probability. It is clearly observed that in the presence of a resetting mechanism, the probability of extreme events is significantly reduced across the entire BA network. This observation is in strong agreement with the conclusion drawn from the Figure 3. Actually, we can clearly derive that the stochastic resetting obviously increase the average occupation across all the nodes in the BA network by comparing the two panels in Figure 5, which corroborates with the results in Figure 4. Besides, we can also intuitively analyze that the probability of EEs occurring in low degree nodes is higher than that in central nodes and nodes adjacent to the center. Further, notice that Equation (13) does not depend on the large scale structure of the topology, and therefore it is conducive to delve into other complex networks through theoretical and simulation analysis.

## 4. Fluctuations in Event Size with Stochastic Resetting

In this section, we focus on the fluctuations in event size in the flux passing through a node with stochastic resetting. To explore how event size fluctuations depend on node connectivity in the presence of stochastic resetting, we analyze the distribution of event sizes with nodes arranged in descending order of their degrees and nodes with the same degree in ascending order of their distance to the resetting node. The visualization of event-size distributions in this order highlights how highly connected nodes (hubs) respond to fluctuations in walker flux compared to peripheral nodes.

**Figure 6:**
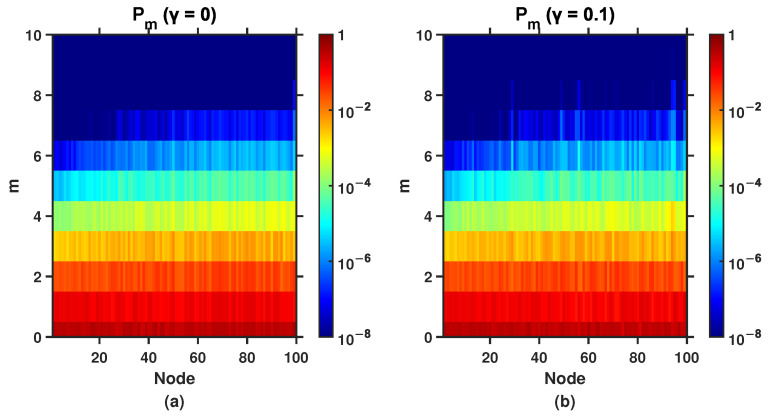
The distribution of event sizes on the BA scale-free network under different resetting rates, with the x-axis representing node number, (**a**) γ=0, (**b**) γ=0.1. Nodes are sorted in descending order of degree, and nodes with the same degree are sorted in ascending order of their distance to the resetting node, and the probability $P_{m}$ is indicated using a color map.

Figure 6 shows the distribution of event size m, measured in units of the standard deviation σ, as a function of node degree and their distance from the resetting node. This theoretical analysis is based on the BA scale-free network used in Figure 4. The color map displays the probability of an event of size *m* occurring on nodes with different degrees or same degrees with different distances to the resetting node. In our research, an event of size m is defined by the condition $m\sigma \leq w-\langle f_{i}\left(r,\gamma \right)\rangle \leq \left(m+1\right)\sigma$, where *w* is the number of walkers discovered on the node. Then, the probability for the occurrence of an event of size m can be described as follows:

(15) Pm=Fm−Fm+1=∑w=qmw=qm+1Wwpi(r,γ)w[1−pi(r,γ)]W−w.

To visualize this theoretical result, we plot the distribution of event sizes as the node degrees and their distances to the resetting node with different resetting rate γ. In Figure 6a, we observe a pronounced asymmetry in the fluctuation landscape: the nodes on the left exhibit a narrow range of event sizes, typically within |m|≤3, and the probabilities for larger event sizes (m>5) rapidly drop below $10^{-6}$. This reflects the fact that hubs receive a relatively stable and high average flux of walkers, making large deviations statistically rare, which leads to the probability for the occurrence of events of large sizes are relatively small. This is consistent with the results shown in Figure 1. The nodes in the middle begin to show moderate broadening in event size distribution. Occasional events up to m≈6 are observed, with probabilities $P_{m}\approx 10^{-6}$, indicating increased flux volatility compared to the hubs. The nodes on the right display the most dramatic fluctuations. Some of these nodes experience extreme events as large as m=7 or more, with non-negligible probabilities (e.g., $P_{6}>10^{-5}$ for certain nodes). This suggests that, although these nodes are rarely visited in the dynamical process performed by random walkers, the few walker arrivals they do receive are sufficient to produce disproportionately large statistical deviations which implies that even occasional visits by a few random walkers contribute to extremely events of large sizes.

Compared to Figure 6a,b demonstrates that event size fluctuations under resetting random walks become more concentrated. Notably, the probability of unit event size (m=1) increases significantly, indicating that stochastic resetting effectively reduces size variability. Furthermore, we observe a marked decrease in extreme events (m>4), consistent with the trends shown in Figure 3. These results collectively demonstrate that resetting suppresses both overall fluctuations and extreme event probabilities.

Interestingly, these effects exhibit degree and distance dependence: while small-degree and peripheral nodes show clear changes in their event size distributions, hubs display less pronounced variations. This suggests that stochastic resetting differentially affects nodes based on their connectivity in BA scale-free networks. Our findings confirm earlier analytical predictions that those nodes with small degrees and far from the resetting node are particularly vulnerable to extreme fluctuations. Furthermore, as for which factor has a greater impact on the event size fluctuations-the size of the degree and the distance to the resetting node, we also tested another sorting method: sorting in ascending order of the distance to the resetting node, with nodes at the same distance sorted in the descending order of their degrees is considered. We found that the result of this sorting method are not significantly different from the former case, meaning that the two factors have little difference in their impact on the event size fluctuations.

The resetting mechanism produces two key effects. First, it diminishes the dominance of central nodes. Second, it makes fluctuations in peripheral regions more discernible while simultaneously mitigating traffic variations network-wide. The specific order visualization in our research reveals these dynamics more clearly, showing a rightward shift in the probability distribution of large events. This spatial redistribution indicates that extreme fluctuations become increasingly concentrated in the network’s periphery, highlighting the systemic vulnerability of low-degree and peripheral nodes under stochastic perturbations.

This analytical approach serves dual purposes: it not only validates previous theoretical predictions but also offers a powerful diagnostic tool for pinpointing fluctuation hotspots and evaluating network resilience in heterogeneous systems.

## 5. Conclusions

This work investigates extreme events occurring on network nodes under stochastic resetting in random walks. We explore how resetting to a central node affects both the probability of extreme events and the fluctuations in event sizes across the network.

Our key finding is that stochastic resetting serves as an effective control mechanism for extreme events in complex networks. The resetting process significantly reduces the probability of EEs across all nodes, and this reduction is particularly pronounced for low-degree nodes and nodes distant from the resetting node, which are naturally more vulnerable to extreme fluctuations in standard random walks.

The physical mechanisms underlying this suppression effect can be understood through several interconnected factors. First, resetting induces a spatial localization effect that concentrates walkers around the resetting node and its neighbors. This localization arises because the resetting process continuously “pulls” walkers back to the designated node, preventing them from accumulating at peripheral nodes where extreme events are most likely to occur in standard random walks. The mathematical structure of the stationary distribution in Equation (10) clearly shows this redistribution, where the resetting term enhances occupation probabilities near the resetting node at the expense of distant nodes.

Second, resetting fundamentally alters the temporal correlation structure of the walker dynamics. In standard random walks without resetting, the exploration process allows for sustained periods where walkers can accumulate at specific nodes through long excursions, leading to large deviations from the mean occupation. Stochastic resetting interrupts these potentially dangerous trajectories by periodically reinitializing the walk, effectively cutting off the paths that would otherwise lead to extreme fluctuations. This temporal regularization reduces the variance in the occupation distribution, as evidenced by the concentration of event sizes around smaller values in Figure 6.

Third, the differential impact on different node types reveals the selective nature of resetting protection. Hub nodes, which naturally attract large but relatively stable fluxes due to their high connectivity, experience modest changes in their fluctuation patterns under resetting. In contrast, peripheral nodes with low degrees show the most dramatic reduction in extreme events because resetting prevents the rare but large influxes that characterize their vulnerability in standard random walk dynamics. This selective protection of the most vulnerable nodes makes resetting particularly valuable of practical applications.

We have demonstrated that resetting not only lowers extreme event probabilities but also concentrates the distribution of event sizes. The fluctuations become more tightly distributed around the mean, with a marked decrease in extreme deviations. This effect arises because resetting redistributes walkers across the network, preventing the accumulation of large fluctuations at any single node.

An important insight from our work is the differential impact of resetting based on both node degree and distance to the resetting node. While all nodes benefit from reduced extreme events, the effect is more substantial for peripheral nodes—those with low degree and located far from the resetting node—compared to hubs or nearby nodes. This suggests that resetting could be particularly valuable for protecting vulnerable nodes in infrastructure networks, where extreme events (such as traffic congestion or overloads) are most likely to occur at poorly connected or remote nodes.

Our analytical framework, validated through numerical simulations, provides a foundation for designing optimal resetting strategies in real-world networks. The results suggest that by carefully tuning the resetting rate γ, network operators can achieve a desired balance between search efficiency (often improved by resetting) and extreme event mitigation. This could have practical applications in designing robust communication networks, transportation systems, and power grids where extreme fluctuations need to be controlled.

Future work could extend these results to other resetting protocols, including multiple resetting nodes [49,52] or resetting biased random walks [24]. The methods developed here may also be applicable to studying extreme events in other stochastic processes with resetting mechanisms.

## Data Availability

Data are contained within the article.

## Abbreviations

The following abbreviations are used in this manuscript:

BA network	Barabási-Albert network
EEs	extreme events
